# Decentralization of snakebite antivenom treatment to indigenous community health centers in the Brazilian Amazon: From demand to the first treatment (the SAVING Program)

**DOI:** 10.1371/journal.pntd.0013011

**Published:** 2025-04-30

**Authors:** Altair Seabra de Farias, Thiago Serrão-Pinto, Deugles Cardoso, Elder Augusto Guimarães Figueira, Fernando Almeida-Val, Tatyana Amorim Ramos, Flávio Santos Dourado, Francisco Edilson Ferreira de Lima-Junior, Lucia Montebello, Manoel Rodrigues Gomes Filho, Leonardo da Silva Ipuchima, Nilzoney Ferreira de Souza, Macio da Costa Arévalo, Marcus Lacerda, Vinícius Azevedo Machado, Felipe Murta, Charles Gerardo, João Vissoci, Fan Hui Wen, Jacqueline Sachett, Wuelton Monteiro

**Affiliations:** 1 School of Health Sciences, Universidade do Estado do Amazonas, Manaus, Brazil; 2 Department of Teaching and Research, Fundação de Medicina Tropical Dr. Heitor Vieira Dourado, Manaus, Brazil; 3 Department of Environmental Surveillance, Fundação de Vigilância em Saúde do Amazonas Dra. Rosemary Costa Pinto, Manaus, Brazil; 4 General Coordination of Zoonoses and Vector-Borne Diseases Surveillance, Ministério da Saúde, Brasília, Brazil; 5 Distrito Sanitário Especial Indígena Alto Rio Solimões, Secretaria Especial de Saúde Indígena, Tabatinga, Brazil; 6 Instituto Leônidas e Maria Deane, Fundação Oswaldo Cruz, Manaus, Brazil; 7 Department of Emergency Medicine, Department of Surgery, Duke University, Durham, North Carolina, United States of America; 8 Duke Global Health Institute, Duke University, Durham, North Carolina, United States of America; 9 Bioindustrial Center, Butantan Institute, São Paulo, Brazil; Lancaster University Faculty of Health and Medicine, United Kingdom of Great Britain and Northern Ireland

## Abstract

Brazilian antivenoms have excellent efficacy in recovering venom-induced coagulopathy, in addition to having a good safety profile with only 10% of patients experiencing a mild reaction such as urticaria or pruritus. More than 3.5 hundred thousand snakebite antivenom vials are produced per year, and all the batches are acquired by the Ministry of Health and distributed free of charge to 2,200 hospitals across the national territory. However, these health facilities are unevenly distributed across the territory, so that the distance a patient needs to travel to receive care is much greater in the Amazon region in comparison to the extra-Amazonia region, leading to a huge access barrier in this region. The lack of access to healthcare facilities for snakebite patients may be greater than 30% in some regions of the Amazonia. The decentralization of SBE treatment with antivenoms to the scope of indigenous community health centers requires the discussion of proper organizational designs and arrangements of practices based on the user needs, singularities of the territory, and the clinical reality of the indigenous populations. In this report, we describe a successful experience of decentralization of antivenom treatment for an indigenous health unit in the Brazilian Amazon, which provides a platform to improve the lives of SBE patients at risk of this life-threatening condition. In this work, we report the experience in the development and implementation of a program to decentralize antivenom treatment for indigenous communities, which represents a significant change in the national policy for snakebite control, with a potential impact on reducing morbidity and mortality from this health problem. In the next steps, SAVING Program will be evaluated through mixed-method studies in regards team and community’s experience within the program, aiming to identify barriers, perceptions about the implementation process, and facilitators for the maintenance/sustainability.

## SBEs in the Brazilian Amazon

In Latin America, the Amazon region has the highest incidence of snakebite envenomations (SBE), with an annual rate of 55 cases per 100,000 inhabitants, which is five times higher than that in the Extra-Amazon region [1]. In the Brazilian Amazon, the annual societal costs of snakebites are around US$8 million, mostly due to the number of working years lost due to ill health, disability, or early death, and treatment costs [2]. In this region, 90% of snakebites are caused by *B. atrox*, leading to systemic and local manifestations [3]. Among systemic complications, systemic bleeding and acute kidney injury stand out, occurring in 15% [4] and 13% [5] of the patients, respectively. Local complications are mainly represented by secondary bacterial infections, in 40% of patients [6]. Despite the lack of robust data on the frequency of long-term disabilities, these are commonly seen in hyperendemic areas of the Brazilian Amazon, especially among indigenous populations, due to the greater probability of secondary bacterial infections, compartment syndrome, and necrosis, whose occurrence is strongly associated with the delay to antivenom treatment [7]. Overall, the case-fatality rate from snakebites in this region ranges from 0.6% in the population living close to urban areas, to 1.4% among indigenous people [8]. Factors associated with increased case-fatality rate are time to medical care >6 hours, age ≥61 years, Indigenous status, and lack of antivenom administration [9]. The main complications associated with death are acute kidney injury, acute respiratory failure, sepsis, circulatory shock, and hemorrhagic or ischemic strokes [9].

## Access to antivenom from the Amazonian indigenous communities

Brazil has four public antivenom producers, the main one being the Butantan Institute [10]. Brazilian antivenoms have excellent efficacy in recovering venom-induced coagulopathy, in addition to having a good safety profile, with only 10% of patients experiencing a mild reaction such as urticaria or pruritus [11]. More than 350,000 snakebite antivenom vials are produced per year, and all the batches are acquired by the Ministry of Health and distributed free of charge to 2,200 hospitals across the national territory [12]. In Brazil, antivenoms are all available in liquid thermolabile formulations, requiring refrigeration from the production laboratory until their administration to the patient. Therefore, a major concern is the lack of an adequate cold chain, which impairs antivenom distribution to remote areas [10]. However, the equipped health facilities are unevenly distributed across the territory, so that the distance a patient needs to travel to receive care is much greater in the Amazon region in comparison to the extra-Amazonia region, leading to a huge access barrier in this region [8,13]. Around 50% of the snakebite patients do not receive any care from the official healthcare system in some regions of the Brazilian Amazonia [7].

Around 1 million self-declared indigenous people live in the Brazilian Amazon, most of them in territories demarcated by the federal government. The number of indigenous territories demarcated in the Brazilian Amazon is 27, each with its respective health district, formed by community health centers [14]. There are no hospitals in these territories, and only basic care is currently provided. In cases of snakebites, for example, the patient only receives basic initial care, with cleaning the lesion and analgesics [8]. Antivenoms are not available in these locations. The consequence of this assistance model results in delayed antivenom administration, with high rates of severe and fatal cases, as well as resistance and reduced trust in the official health system [8,15,16]. In previous surveys, we found an unexpectedly high underreporting of snakebite cases and associated deaths in some riverine and indigenous communities [7]. Geographic and cultural barriers were mentioned as reasons for not seeking care in such cases, and opting to treat with indigenous medicines only [17]. Also, a large degree of fragmentation was observed in the itineraries to care, marked by several changes in means of transport and waiting times along the route, which can take days to complete [18]. Additionally, disabilities are also important outcomes to consider among indigenous populations in such scenarios [3].

The decentralization of SBE treatment with antivenoms to indigenous community health centers requires the discussion of proper organizational designs and arrangements of practices based on the user needs, singularities of the territory, and the clinical reality of the indigenous populations. In this report, we describe a successful experience of decentralization of antivenom treatment for indigenous health units in the Brazilian Amazon (SAVING Program), which provides a platform to improve the lives of SBE patients at risk of this life-threatening condition.

## The national policy for healthcare for indigenous peoples and the SAVING Program

The SAVING Program is a component of the National Policy for Healthcare for Indigenous Peoples, which adopts complementary and tailored models for organizing services aimed at protecting, promoting, and restoring the health of this population, through the Indigenous Health Secretariat of the Brazilian Ministry of Health (BMoH) [19]. Within the scope of this policy, the SAVING Program creates a network of services on indigenous territories to overcome deficiencies in coverage, access, and acceptability of healthcare for indigenous victims of snakebites. The program was possible only after the improvement of the capacity of health units, by applying the principles of decentralization, universality, equity, community participation, and social control.

The starting point for an antivenom decentralization program began in 2019, when, through a joint document, the indigenous leaders of the Indigenous Health Districts of the Amazonas state, demanded to the health authorities antivenoms to provide treatment for SBEs in the community health centers. The *Fundação de Vigilância em Saúde Dra. Rosemary Costa Pinto* (FVS-RCP), the institution responsible for SBE surveillance and AV distribution in the state, reported the situation to the Venomous Animals Technical Group and to the Indigenous Health Secretariat of the BMoH. At that time, the stocks of antivenom vials provided by the Ministry of Health to the state of Amazonas were insufficient to expand the network of units registered to treat snakebites. In addition, this organization was faced with a demand not covered by the National Program for the Control of Accidents caused by Venomous Animals, which structured an antivenom treatment policy historically centered on hospitals.

Even though the BMoH signaled that a decentralization program would not be possible to be implemented in the short term, a technical group was formed to build a culturally and logistically viable plan. This group was constituted by FVS-RCP (coordination), Venomous Animals Technical Group and Indigenous Health Secretariat of the BMoH, *Fundação de Medicina Tropical Dr. Heitor Vieira Dourado* (FMT-HVD, tertiary hospital serving as reference for snakebite treatment in the Amazonas state), representatives of the Indigenous Health Special Secretariat of the BMoH and of the seven Special Indigenous Health Districts, and experts of the Butantan Institute (the major antivenom producer in Brazil) and of the University of São Paulo. The first technical group meeting was held in Manaus, on February 24–28, 2020.

**Fig 1:**
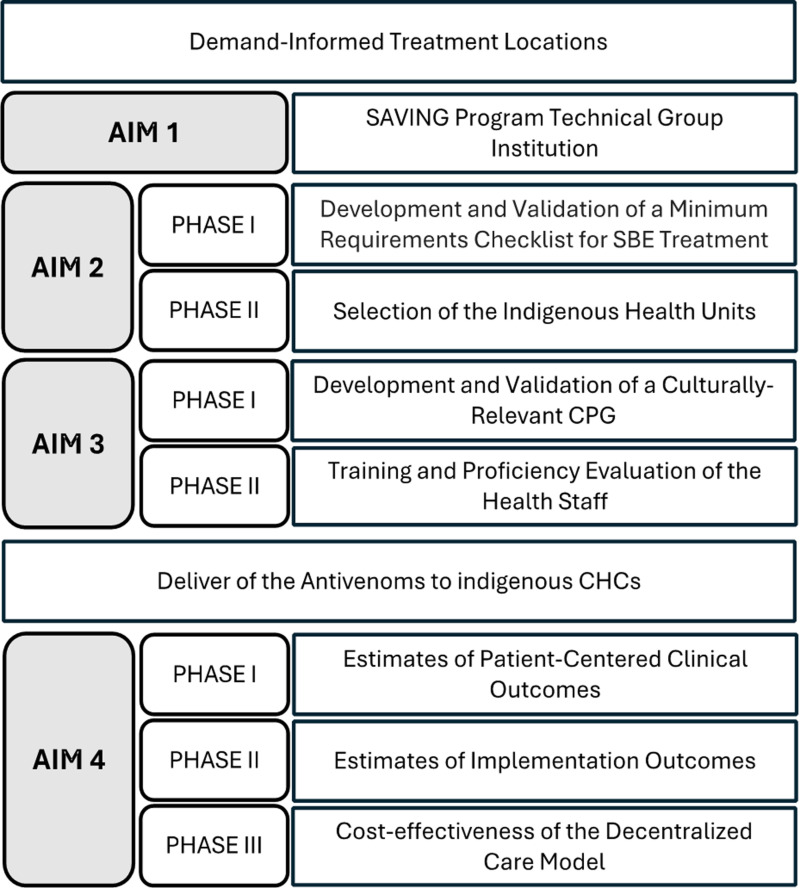
Aims of the SAVING (*S*nakebite *A*nti*V*enom *I*mplementation *N*eed to Be *G*uaranteed) Program.

In this meeting, a multimodal decentralization plan named SAVING (*S*nakebite *A*nti*V*enom *I*mplementation *N*eed to Be *G*uaranteed) was discussed, as presented in Fig 1.

We should mention here in what temporal context the first SAVING technical group meeting was held. On February 16, 2020, the first case of COVID-19 in Brazil was confirmed in São Paulo, a 61-year-old man with a history of traveling to Italy [20]. On March 11, the WHO states that COVID-19 is characterized as a pandemic [21]. On March 20, the BMoH declared community transmission throughout the country and established that infected people and residents of the same household must be isolated [22]. On March 13, the state of Amazonas had its first confirmed case of COVID-19, in Manaus [23]. A few months later, Manaus would stand out as the epicenter of COVID-19 transmission in Brazilian Amazonia, with a collapse of the health system and a steep increase in mortality rates [24].

The Amazonas Bipartite Intermanagerial Commission (CIB/AM), which is the collegiate body within the Unified Health System that agrees on the management and execution process of actions and services at the state and municipal levels, approved the implementation of the decentralization of antivenom treatment to indigenous health centers in the state of Amazonas [25].

Still in March 2020, temporary measures were taken to prevent the entry of COVID-19 into indigenous areas, by establishing protective sanitary barriers to control the transit of people and goods heading to these areas [26]. To this end, the National Indian Foundation suspended the granting of new authorizations to enter indigenous lands, apart from those necessary to continue providing essential services to communities. In the context of this crisis, the SAVING Program had its activities dependent on the entry and exit of people in indigenous areas resumed after the end of the public health emergency, by the end of April 2022 [27]. During this period, planning activities and others that could be carried out through virtual meetings were maintained.

## Selection of the health units

One barrier identified by members of the Ministry of Health is the lack of a strategic stock to supply antivenoms for new units. In 2013, new sanitary rules from the Brazilian Health Regulatory Agency required laboratories to improve good manufacturing practices (GMP). Instituto Butantan was the first national manufacturer to obtain a GMP certification for production and is currently the only manufacturer providing antivenoms to the BMoH [28]. At this stage, the technical group settled that the decentralization would start only in two indigenous health centers in each of the seven Indigenous Districts of the Amazonas state, that is, 14 (5%) of a total of 282 indigenous health units in the Amazonas state.

Indigenous Districts were contacted to provide the technical group a list of indigenous health centers to be selected to provide antivenom treatment in the SAVING Program. Only community health centers operating with a 24-hour electricity network, and with backup generators (for safe storage of liquid antivenoms), and full-time presence of physicians to prescribe and supervise antivenom treatment, were eligible. The selection of units would still consider health center spatial and populational coverage, the number of SBEs in the health center’s coverage area in the last 3 years, and ability and time to transport patients to a hospital providing antivenom. Since there was no evidence-based method to evaluate the capacity of health units for antivenom treatment, nor what the absolute minimum supplies and staff are necessary for safe and effective antivenom administration and clinical management, the technical group recommended the development and validation of a checklist for this purpose. The experts proposed that the treatment of patients with mild and moderate clinical conditions could be carried out in indigenous health units, but in the event of progression to severity, the patient would receive antivenom in the community and later be transferred to the hospital for continued management. To achieve this, the health unit should have availability of 78 essential items, including human resources, equipment, supplies, and medicines to be accredited to perform antivenom treatment as proposed above [29].

The checklist was applied to the units indicated by the Indigenous Districts, and the conclusion was that community health centers in indigenous territories have high potential to provide safe and effective antivenom treatment [30]. After that, a representative from FVS-RCP visited each of the 14 selected units to certify that the units were able to start treating SBE patients with antivenom.

## Training health staff to antivenom treatment

The BMoH has an official guideline with general recommendations for the management of SBEs [31]. This guideline presents the general clinical aspects of SBE and antivenom treatment centered in hospitals. The guideline does not address pre-hospital care and first aid; antivenom storage, preparation, and administration; wound care; ancillary treatment of different local and systemic manifestations; referral to higher complexity healthcare services; discharge criteria; clotting time procedure; and reporting of cases to epidemiological surveillance systems. Furthermore, the SAVING technical group identified the need for a guideline that considered the experience of academic experts and health specialists with professional experience in the Amazon region, including doctors and nurses working in indigenous health districts. This clinical practice guideline, which was culturally adapted for indigenous health units [32], was used as training material for health professionals before starting antivenom treatment in the units. Trainings included emergency care, diagnosis and severity classification of the snakebites, performing of the clotting time test, preparation of antivenom for administration, antivenom administration, wound care, patient follow-up, referral of severe snakebites to a hospital, recognition of snakebite complications, patients’ discharge and outpatient follow-up, case reporting, and antivenom storage.

From June 27 to July 1, 2022, the first training and proficiency evaluation of the healthcare staff based on indigenous units was carried out at FMT-HVD, in Manaus. One physician and one nurse from each of the 14 community health centers participated in the training, with a total of 28 trainees. After a 24-hour round of training using the validated formative package, a test was administered. The correction of the test allowed the SAVING team to return to delve deeper into the topics on which the trainees performed less well.

Focus group discussions carried out with these professionals demonstrated that the health staff are very receptive, open to learn and personally invested in improving SBE management and replicate the information received in training with other team members [12,17]. Furthermore, the engagement with indigenous caregivers (*pajés*, the indigenous shamans) proved to be a possible strategy for the timely referral of SBE patients to a facility equipped with antivenoms in a comprehensive care process [7]. The barriers identified in this process of co-construction were a lack of training in SBE treatment during technical or university courses and concerns about the turnover of human resources and irregular antivenom and medicines supply in the health units [17].

Printed and digital copies of the care package guidelines were provided to the professionals. They were asked to share these copies with their colleagues. The technical group verified that an instant messenger software (WhatsApp) was used by all training participants, and that is why this messaging application was chosen as the communication channel between professionals working in indigenous areas and SAVING specialists. The objective is to offer a channel to clarify doubts about the management of snakebite cases. SMS and telephone calls were communication alternatives also made available to professionals.

## The first antivenom vials in the indigenous health units

In December 2021, a transfer agreement for 700 vials of *Bothrops* antivenom and 100 vials of *Bothrops-Lachesis* antivenom was signed between the Butantan Institute and FMT-HVD. According to this agreement, these vials would be used to begin the implementation of the SAVING Program, under the supervision of the FVS-RCP, until the BMoH maximizes the inventory of antivenoms for the state of Amazonas. With this transfer, SAVING Program was piloted in three indigenous health poles: Belém do Solimões and Vendaval poles, in the Upper Solimões River Indigenous District, and the Pari-Cachoeira pole, in the Upper Negro River Indigenous District. The transport of antivenoms to the poles of the Upper Solimões River Indigenous District was carried out by plane to the municipality of Tabatinga, and then by boat to the health units. For the Pari-Cachoeira pole, transportation was done by plane. These centers were chosen because they were immediately prepared to start antivenom treatment, as per the previous application of the verification checklist, and because they had a high burden of snakebites. Belém do Solimões health pole received the antivenom vials on November 22, 2022, Pari-Cachoeira on February 7, 2023, and Vendaval on May 17, 2023. The antivenom delivery visit was accompanied by an 8-hour retraining.

For the antivenom inventory for the year 2024, the BMoH increased the quota of antivenom vials purchased from the Butantan Institute for the state of Amazonas, and currently all the 14 units included in the SAVING Program are providing antivenom. In the Middle Solimões River Indigenous District, the Eirunepé pole was replaced by the Cuiú-Cuiú pole, since the power generator at the first pole was not operating. On April 23–24, 2024, health professionals at these centers were retrained in an online course. Doctors, nurses, nursing assistants, and pharmacists from each center were trained. After the training, antivenom was delivered to the units and the group is currently monitoring the effectiveness and feasibility of the program.

**Table 1** shows the locations of the indigenous community centers selected to the SAVING Program.

**Table 1:** Locations of the indigenous community centers selected to the SAVING Program.

Indigenous Health District	Health Pole
Médio Rio Solimões Afluetes	Cuiú-Cuiú
	Biá
Médio Rio Purus	Marrecão
	Crispim
Alto Rio Negro	Uiaurete
	Pari-Cachoeira
Alto Rio Solimões	Solimões
	Vendaval
Manaus	Boca do Jauari
	Kwatá
Parintins	Umirituba
	Nova Esperança
Vale Do Javari	Polo Base Médio Ituí
	Médio Curuçá

## The first antivenom treatment in an indigenous health unit

On December 1, 2022, the first case treated by the SAVING Program was an 8-year-old girl of Tikuna ethnicity and resident in the Novo Cruzador Village, which is served by the Belém do Solimões health pole. She was bitten on her left foot when she was playing with other children in a wooded area near her house. Family members applied salt and crushed herbs to the bite site and waited for symptoms’ improvement. The health team was informed of the case by the child’s neighbors because the parents did not want to seek medical attention despite the child’s clinical condition becoming critically ill. A nurse and a nursing assistant went to the village, on a 2-hour route combining a part by boat and a walk through the forest, supervised by a physician by phone. They found the girl in her house, in severe condition, in intense pain, edema over the entire length of the bitten limb, bruising and bleeding from a recent cut on her finger, gum bleeding, and hematuria. The girl was very pale and prostrated. Given the possible resistance of the child’s parents to taking the girl to the health unit, the professionals carried the antivenom with them to the village in a cooler box with reusable ice packs. Given the severity of the case, twelve vials (total of 120 mL) of *Bothrops* antivenom were administered, 20 min after premedication with IV hydrocortisone (500 mg) and oral dexchlorpheniramine (5 mg).

The AV treatment was administered 48 hours after the bite. Immediately after administering the AV, the professionals transported the patient to the health center, placing her lying down in a hammock. At the center, she was kept under intravenous hydration, and intravenous metamizole was given on demand for pain. Clotting time was normal 12 hours after antivenom administration. No early adverse reactions were reported. The patient developed a secondary bacterial infection and was treated with IV ceftriaxone. Two days after admission, the patient was discharged without complaints. This case illustrates several aspects of SBE treatment in these populations.

At the health center, the nurse responsible for the child’s care, impressed by the rapid effect of the antivenom, sent the following message to the SAVING group:

*“Let me tell you this one. The little girl was about to find out almost 48 hours after the bite. We found out later in the afternoon, when they informed me, and I sent the team there because the father didn’t want to bring her to the health post. A mess. We took the antivenom, 12 vials, and went to the girl’s house. When we got there, she was in severe condition, bleeding from everywhere. On the finger, in a cut, which he had about 3 days before. Just after she had already taken the antivenom, the 12 vials, the bleeding stopped. The team was all amazed. We are following her here at the health post.”*

## Final remarks

Antivenom treatment is very effective and has a safety profile that allows it to be carried out in community health centers. We report the experience in the development and implementation of a program to decentralize antivenom treatment for indigenous communities, which represents a significant change in the national policy for snakebite control, with a potential impact on reducing morbidity and mortality from this health problem. In the next steps, SAVING Program will be evaluated through mixed-method studies in regards team and community’s experience within the program, aiming to identify barriers, perceptions about the implementation process, and facilitators for sustainability.

### Ethical statement

This protocol was submitted and approved by the National Research Ethics Commission (approval number 4,993,083/2020).
